# 3,4-Difluorobenzocurcumin Inhibits Vegfc-Vegfr3-Erk Signalling to Block Developmental Lymphangiogenesis in Zebrafish

**DOI:** 10.3390/ph14070614

**Published:** 2021-06-26

**Authors:** Kazuhide S. Okuda, Mei Fong Ng, Nur Faizah Ruslan, Neil I. Bower, Dedrick Soon Seng Song, Huijun Chen, Sungmin Baek, Philip S. Crosier, Katarzyna Koltowska, Jonathan W. Astin, Pei Jean Tan, Benjamin M. Hogan, Vyomesh Patel

**Affiliations:** 1 Organogenesis and Cancer Program, Peter MacCallum Cancer Centre, Melbourne, VIC 3000, Australia; Ben.Hogan@petermac.org; 2 Sir Peter MacCallum Department of Oncology, University of Melbourne, Melbourne, VIC 3000, Australia; 3 Department of Anatomy and Physiology, University of Melbourne, Melbourne, VIC 3000, Australia; 4 Division of Genomics of Development and Disease, Institute for Molecular Bioscience, The University of Queensland, St Lucia, QLD 4072, Australia; n.bower@imb.uq.edu.au (N.I.B.); h.chen@imb.uq.edu.au (H.C.); sungminbaek@gmail.com (S.B.); kaska.koltowska@igp.uu.se (K.K.); 5 Cancer Research Malaysia, Subang Jaya 47500, Selangor, Malaysia; mfong.ng@gmail.com (M.F.N.); faizah.ruslan@cancerresearch.my (N.F.R.); dedrick.song@cancerresearch.my (D.S.S.S.); tanpeijean@gmail.com (P.J.T.); vpatel.edit@gmail.com (V.P.); 6 Stowers Institute for Medical Research, Kansas City, MO 64110, USA; 7 Department of Molecular Medicine & Pathology, School of Medical Sciences, The University of Auckland, Auckland 1010, New Zealand; ps.crosier@auckland.ac.nz (P.S.C.); j.astin@auckland.ac.nz (J.W.A.); 8 Department of Immunology, Genetics and Pathology, Uppsala University, 751 85 Uppsala, Sweden

**Keywords:** 3,4-Difluorobenzocurcumin, zebrafish, lymphatic, Vegfc, Vegfr3, Erk

## Abstract

Lymphangiogenesis, the formation of new lymphatic vessels from pre-existing vasculature, plays critical roles in disease, including in cancer metastasis and chronic inflammation. Preclinical and recent clinical studies have now demonstrated therapeutic utility for several anti-lymphangiogenic agents, but optimal agents and efficacy in different settings remain to be determined. We tested the anti-lymphangiogenic property of 3,4-Difluorobenzocurcumin (CDF), which has previously been implicated as an anti-cancer agent, using zebrafish embryos and cultured vascular endothelial cells. We used transgenic zebrafish labelling the lymphatic system and found that CDF potently inhibits lymphangiogenesis during embryonic development. We also found that the parent compound, Curcumin, does not inhibit lymphangiogenesis. CDF blocked lymphatic and venous sprouting, and lymphatic migration in the head and trunk of the embryo. Mechanistically, CDF impaired VEGFC-VEGFR3-ERK signalling in vitro and in vivo. In an in vivo pathological model of Vegfc-overexpression*,* treatment with CDF rescued endothelial cell hyperplasia. CDF did not inhibit the kinase activity of VEGFR3 yet displayed more prolonged activity in vivo than previously reported kinase inhibitors. These findings warrant further assessment of CDF and its mode of action as a candidate for use in metastasis and diseases of aberrant lymphangiogenesis.

## 1. Introduction

Lymphatic vessels play important roles that include maintenance of tissue fluid homeostasis, facilitation of immune responses, and dietary fat absorption [1]. Aberrant lymphatic growth is observed in many human diseases including cancer, lymphatic malformation and chronic inflammation and its inhibition has resulted in alleviation of pathological symptoms in many of these diseases [2,3,4]. Despite this, there are only a few FDA-approved drugs that target lymphangiogenesis and their efficacy against diseases of increased lymphangiogenesis remains to be seen, emphasizing the urgent need for novel lymphatic modulatory agents [3,5]. To this end, humanized monoclonal antibodies VGX-100 and IMC-3C5 that target the Vascular endothelial growth factor C (VEGFC)/VEGF receptor 3 (VEGFR3) pathway essential for lymphangiogenesis, are undergoing clinical evaluation as novel cancer therapeutics (NCT01514123 and NCT01288989) [6]. Given the mixed results of recent trials, it is likely that multiple or combinations of therapeutics may be needed for maximal efficacy in different lymphatic presentations [5,7].

Curcumin, a dietary compound from turmeric has been suggested to impair tumour progression by modulating various pathways involved in cell cycle progression, inflammation, apoptosis, and angiogenesis, that are essential for tumour growth and metastasis [8]. Curcumin has also been suggested to inhibit lymphangiogenesis in VEGFC-mediated matrigel plug assay and in gastric cancer cells xenotransplanted in mice via inhibition of VEGFR2 and VEGFR3 protein and mRNA levels [9,10]. A recent study had suggested that curcumin also inhibits the mRNA and protein levels of VEGFD, an alternative ligand of VEGFR3 essential for tumour-associated lymphangiogenesis [11,12]. However, curcumin suffers from poor bioavailability due to unfavourable water solubility, ease of degradation/metabolism and limited absorption when taken orally [13]. 3,4-Difluorobenzocurcumin (CDF) is a curcumin analogue generated by an addition of a difluorobenzylidene moiety to the curcumin core structure [14]. The resulting compound was shown to have significantly increased bioavailability and anti-cancer properties when compared with the parental compound curcumin [15,16,17,18,19]. CDF has been shown to inhibit tumour progression by modulating multiple molecular targets including Phosphatase and tensin homolog (PTEN), Enhancer of zeste homolog 2 (EZH2), and miRNAs such as miR-21 and miR-101 [15,18]. Although CDF holds promise as a novel anti-cancer therapeutic, its potential as an anti-lymphangiogenic drug remains unexplored.

Zebrafish have proven a useful model for lymphatic research as they possess a functional lymphatic system with conserved molecular mechanisms controlling lymphangiogenesis and lymphatic specification (Reviewed in detail in [20,21]). Of note, real time visualisation and quantification of lymphatic development can be performed using zebrafish due to the availability of transgenic lines that fluorescently label lymphatic vessels such as the *lymphatic endothelial hyaluronan receptor 1b* (*lyve1b*) and *prospero homeobox 1a* (*prox1a*) reporter lines [22]. Taking advantage of this, the zebrafish model had been used to identify novel anti-lymphangiogenic drugs that can be translated to mammalian settings [23,24]. Furthermore, a recent study has utilised the zebrafish model to identify an effective therapy for a patient with complex lymphatic anomaly with gain of function mutation in the A-Raf proto-oncogene, serine/threonine kinase (ARAF) gene [25].

In the current study, we took advantage of zebrafish models to investigate the anti-lymphangiogenic activity of CDF. We find that CDF is a potent anti-lymphangiogenic agent in contrast to Curcumin which demonstrated minimal anti-lymphangiogenenic activity. We show that CDF inhibits VEGFC-induced phosphorylation of ERK in vitro, and venous endothelial phosphorylation of Erk in zebrafish embryos. CDF inhibits excessive proliferation and pathological endothelial cell phenotypes caused by *vegfc* overexpression in zebrafish, further indicating that CDF targets the Vegfc/Vegfr3/Erk pathway. Unlike the VEGFR kinase inhibitor sunitinib malate (SM), CDF does not inhibit VEGFR3 kinase activity. CDF shows prolonged anti-lymphangiogenic activity in vivo even following brief treatments. Thus, our data suggest that CDF is a potent anti-lymphangiogenic agent that may hold promise as a novel therapeutic for lymphatic-associated diseases.

## 2. Results

### 2.1. CDF Inhibits Lymphangiogenesis in Zebrafish

**Figure 1:**
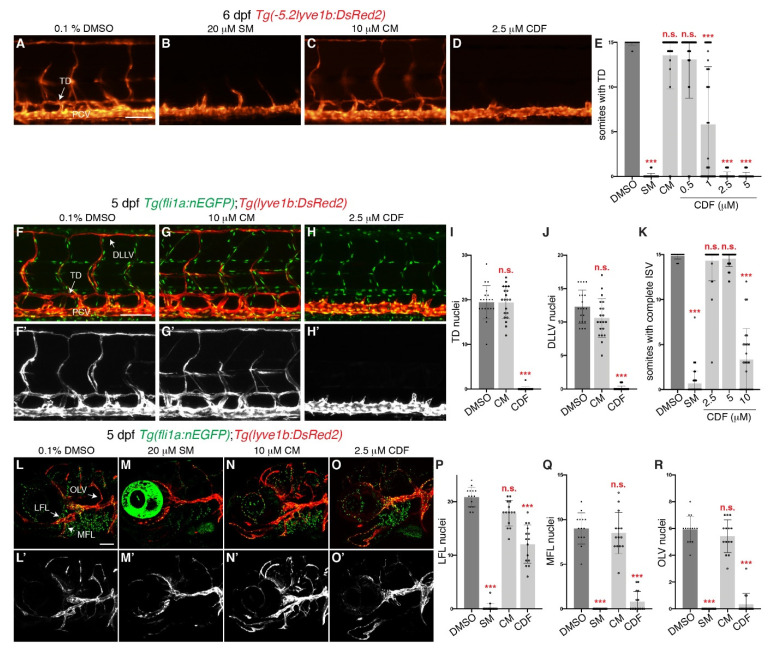
3,4-Difluorobenzocurcumin treatment inhibits trunk and facial lymphatic development in zebrafish. (**A**–**D**) Lateral fluorescent images of 6 dpf *Tg(-5.2lyve1b:DsRed2)* larvae treated with either 0.1% DMSO (**A**), 20 μM sunitinib malate (SM, **B**), 10 μM curcumin (CM, **C**), or 2.5 μM 3,4-Difluorobenzocurcumin (CDF, **D**). CDF inhibits lymphatic development in a dose-dependent manner in zebrafish. (**E**) Quantification of somites with thoracic duct (TD) tissue fragments in 6 dpf *Tg(-5.2lyve1b:DsRed2)* larvae treated with either 0.1% DMSO (n = 49 larvae), 20 μM SM (n = 51 larvae), 10 μM CM (n = 32 larvae), or CDF at 0.5 μM (n = 34 larvae), 1 μM (n = 37 larvae), 2.5 μM (n = 48 larvae), or 5 μM (n = 46 larvae). (**F**–**H’**) Lateral confocal images of 5 dpf *Tg(fli1a:nEGFP);Tg(-5.2lyve1b:DsRed2)* larvae treated with either 0.1% DMSO (**F**,**F’**), 10 μM CRM (**G**,**G’**), or 2.5 μM CDF (**H**,**H’**). Images (**F’**–**H’)** represent the *Tg(-5.2lyve1b:DsRed2)* expression of images (**F**–**H)**. (**I,J**) Quantification of TD (**I**) or dorsal longitudinal lymphatic vessel (DLLV, J) nuclei across 9 somites in 5 dpf *Tg(fli1a:nEGFP);Tg(-5.2lyve1b:DsRed2)* larvae treated with either 0.1% DMSO (n = 20 larvae), 10 μM CM (n = 20 larvae), or 2.5 μM CDF (n = 24 larvae). (**K**) Quantification of somites with intersegmental vessels (ISVs) in 48 hpf *Tg(fli1a:EGFP)* embryos treated with either 0.1% DMSO (n = 47 embryos), 20 μM SM (n = 56 embryos), or CDF at 2.5 μM (n = 35 embryos), 5 μM (n = 39 embryos), or 10 μM (n = 27 embryos). CDF at 2.5 μM does not inhibit primary angiogenesis. Representative fluorescent images of graph K can be found in Figure S2A–E. (**L–O’**) Lateral confocal images of *Tg(fli1a:nEGFP);Tg(-5.2lyve1b:DsRed2)* larvae treated with either 0.1% DMSO (**L**,**L’**), 20 μM SM (**M**,**M’**), 10 μM CM (**N**,**N’**), or 2.5 μM CDF (**O**,**O’**). CDF at 2.5 μM blocks facial lymphatic development. Images (**L’–O’**) represent the *Tg(-5.2lyve1b:DsRed2)* expression of images (**L–O**). (**P**–**R**) Quantification of lateral facial lymphatic (LFL, **P**), medial facial lymphatic (MFL, **Q**), or otolithic lymphatic vessel (OLV, **R**) nuclei in 5 dpf *Tg(fli1a:nEGFP);Tg(-5.2lyve1b:DsRed2)* larvae treated with either 0.1% DMSO (n = 14 larvae), 20 μM SM (n = 16 larvae), 10 μM CM (n = 14 larvae), or 2.5 μM CDF (n = 15 larvae). Statistical test: Kruskal-Wallis test were conducted for graphs (**E**,**I**–**K**,**P**–**R**). PCV: posterior cardinal vein. *p* ≤ 0.001 (***) and n.s. indicates not significant. Scale bars: 100 μm.

To determine if CDF has anti-lymphangiogenic properties, we utilized the *Tg(lyve1b:DsRed2)* zebrafish transgenic line, which fluorescently labels lymphatics and veins [26] (Figure 1A). Treatment of 24 h post fertilisation (hpf, before lymphatic sprouting commenced [27]) *Tg(lyve1b:DsRed2)* embryos with CDF inhibited thoracic duct (TD) formation in a dose-dependently manner as scored at 6 days post-fertilisation (dpf) (Figure 1A,D,E). The minimum concentration required for CDF to disrupt TD formation was 1 μM, while complete inhibition was observed at 2.5 μM, comparable to that observed in larvae treated with 20 μM SM, a FDA-approved small molecule inhibitor of VEGFR kinase activity [28] (Figure 1B,D,E). Hence, we chose to use the dose of 2.5 μM for subsequent experiments. Interestingly, despite the previously reported anti-lymphangiogenic activity of curcumin [9,10,11,29], treatment with up to 10 μM curcumin failed to inhibit TD formation (Figure 1C,E), while treatment with higher concentrations was toxic (≥20 μM, data not shown). Quantification of lymphatic endothelial cell (LEC) numbers in trunk lymphatic vessels, has been shown to be a very accurate measure of lymphatic development in zebrafish [30,31]. Therefore, we examined how CDF and/or curcumin at 2.5 μM and 10 μM respectively, impact upon TD and dorsal longitudinal lymphatic vessel (DLLV) LEC numbers. Treatment with CDF strongly reduced TD and DLLV LEC numbers when compared to DMSO-treated larvae at 5 dpf (Figure 1F,F’,H–J). Treatment with curcumin at 10 μM had no impact on TD and DLLV LEC numbers (Figure 1G,G’,I,J). To exclude the possibility that CDF only inhibits trunk lymphangiogenesis, we also examined facial lymphatic development. Treatment with 20 μM SM reduced the LEC number of facial lymphatic vessels such as the lateral facial lymphatic (LFL), medial facial lymphatic (MFL), and the otolithic lymphatic vessel (OLV), while treatment with 10 μM curcumin did not alter LEC numbers for these facial lymphatic vessels when compared to DMSO (Figure 1L–N’,P–R). Treatment with 2.5 μM CDF significantly reduced MFL and OLV LEC number when compared to DMSO-treated larvae (Figure 1O,O’,Q,R). Interestingly, LEC number in the LFL was significantly reduced but not completely inhibited in larvae treated with CDF (Figure 1O–P). CDF treatment also results in impaired anterior cranial protrusion (Figure S1A–C,E,F, red arrows) and mildly delayed pharyngeal cartilage development (Figure S1F, yellow arrow). While it is possible that development of some facial lymphatic vessels (for example the lymphatic branchial arches) may be affected, there were no gross morphological defects in the craniofacial cartilages alongside which the LFL, MFL and OLV form (Figure S1F, purple arrows). Therefore, these craniofacial defects alone would not explain the potent impairment of facial lymphangiogenesis in CDF-treated larvae.

Common signalling pathways regulate angiogenesis and lymphangiogenesis [20], and thus anti-lymphangiogenic agents may also be anti-angiogenic (eg. SM [28]). To investigate if CDF inhibits Vegfa/Vegfr2 (Kdr/Kdrl) pathway-dependent angiogenic sprouting of intersegmental vessels (ISVs) from the dorsal aorta [32]), we treated 16 hpf *Tg(fli1a:EGFP)* embryos with CDF and observed inhibited ISV formation at 10 μM but not 5 μM or 2.5 μM (Figure 1K, Figure S2A–E). ISV sprouts were still visible in embryos treated with 10 μM CDF but not with SM (Figure S2B,E). In comparison, the lower dose of 2.5 μM of CDF blocked lymphangiogenesis selectively (Figure 1E,I,J). Therefore, CDF is a more potent inhibitor of lymphangiogenesis than angiogenesis.

### 2.2. CDF Inhibits Lymphatic and Venous Sprouting and Lymphatic Endothelial Cell Migration in Zebrafish

To shed light upon the mechanism by which CDF inhibits lymphangiogenesis, we sought to investigate the exact developmental stages affected. We examined secondary sprout formation in zebrafish (endothelial sprouting from the posterior cardinal vein (PCV)), which depends on Vegfc/Flt4 (zebrafish orthologue of VEGFR3) signalling [33,34,35]. As expected, treatment with 20 μM SM inhibited all secondary sprout formation at 36 hpf (Figure S2F,G,I). Treatment with 2.5 μM CDF also attenuated secondary sprout formation (Figure S2H,I). We next tested whether CDF treatment inhibits formation of parachordal LECs (PLs), the first LECs to sprout from the PCV and migrate to the horizontal myoseptum (HM) [27]. 2.5 dpf embryos treated with either SM or CDF had significantly reduced PLs (Figure S2J–M). Overall, these results show that CDF can inhibit lymphatic and venous sprouting to perturb lymphatic development in zebrafish.

**Figure 2:**
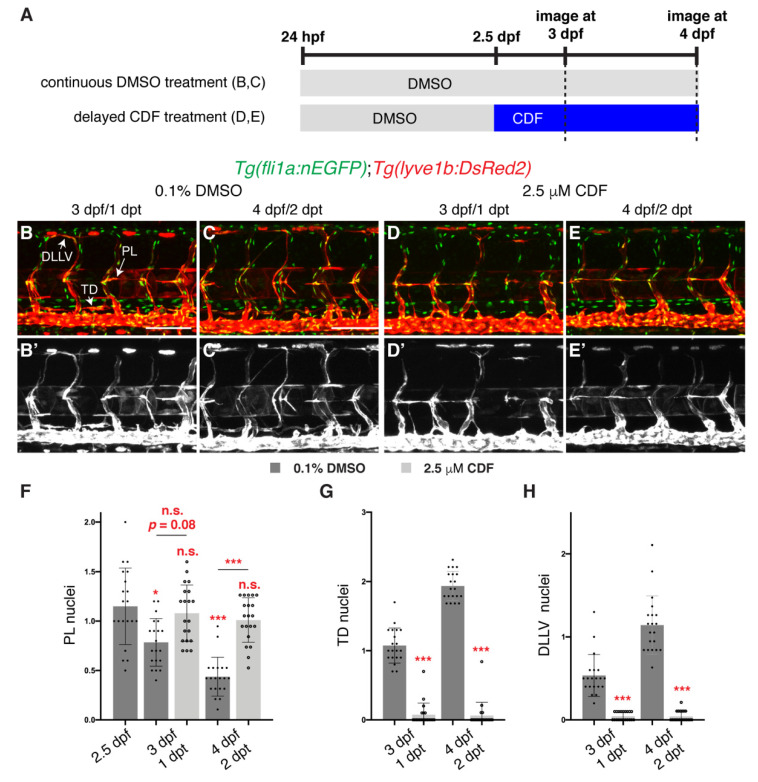
3,4-Difluorobenzocurcumin treatment inhibits lymphatic migration. (**A**) Schematic representation of the treatment schedule for larvae in images (**B**–**E’**). (**B**–**E’**) Lateral confocal images of 3 (1 days post-treatment (dpt), **B**,**B’**,**D**,**D’**) and 4 (2 dpt, **C**,**C’**,**E**,**E’**) dpf *Tg(fli1a:nEGFP);Tg(-5.2lyve1b:DsRed2)* larvae treated from 2.5 dpf with either 0.1% DMSO (**B**–**C’**) or 2.5 μM 3,4-Difluorobenzocurcumin (CDF, **D**-**E’**). LEC migration is stalled in larvae treated with CDF. Images (**B’**–**E’**) represent the *Tg(-5.2lyve1b:DsRed2)* expression of images (**B**–**E**). (**F**–**H**) Quantification of parachordal LECs (PL, **F**), thoracic duct (TD, **G**) or dorsal longitudinal lymphatic vessel (DLLV, **H**) nuclei per somite in *Tg(fli1a:nEGFP);Tg(-5.2lyve1b:DsRed2)* larvae treated with either 0.1% DMSO or 2.5 μM CDF at indicated timepoints (n = 20 embryos/larvae). Statistical test: Kruskal-Wallis test was conducted for graph F and Mann-Whitney test were conducted for graphs G and H. *p* ≤ 0.001 (***), *p* ≤ 0.05 (*), n.s. indicates not significant. Scale bars: 100 μm.

We next investigated whether CDF inhibits the ongoing migration of LECs after they have sprouted out from the vein, a process that is also dependent on the Vegfc/Flt4 signalling pathway [36]. We treated 2.5 dpf embryos with CDF, when PLs have formed [27] (Figure 2A). During normal development, PLs initially found in the HM progressively decrease in number as they depart the HM along their migratory paths (Figure 2B–C’,F), while LEC number in the developing TD and the developing DLLV concurrently increase (Figure 2B–C’,G,H). In comparison, PLs in larvae treated with CDF remained stuck in the HM at 3 and 4 dpf (Figure 2D–F) and displayed reduced LEC numbers in the TD and DLLV (Figure 2D–E’,G,H). Overall, these results show that CDF can inhibit both lymphatic sprouting and migration to perturb lymphatic development in zebrafish.

### 2.3. CDF Inhibits VEGFC-Induced ERK Signalling in Human Endothelial Cells In Vitro and Zebrafish Endothelial Cells In Vivo

**Figure 3:**
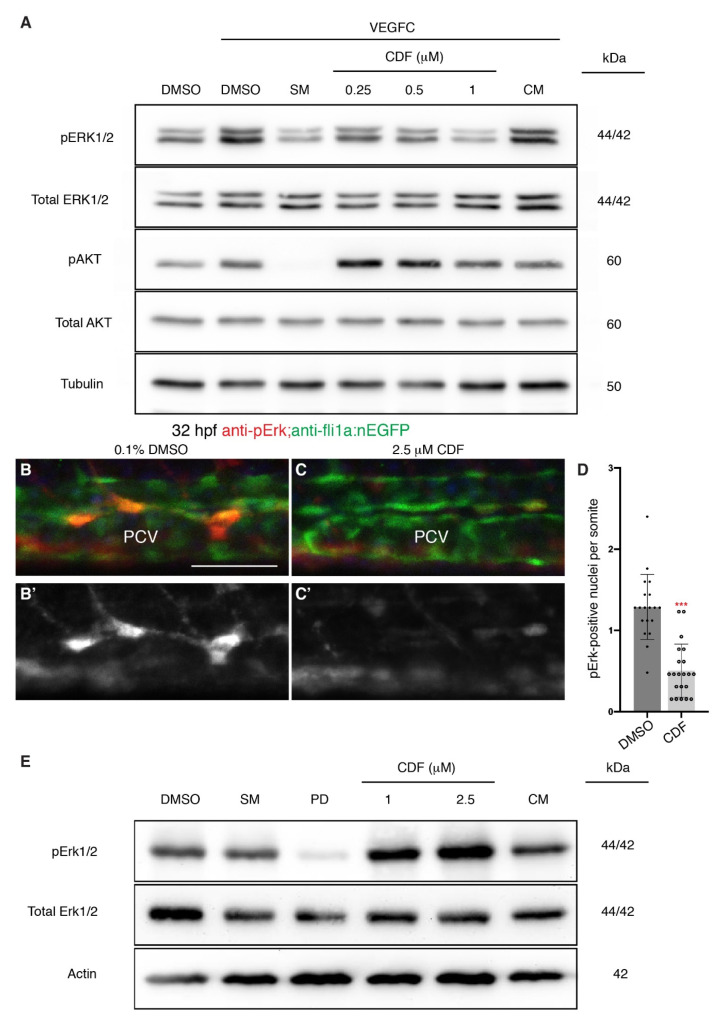
3,4-Difluorobenzocurcumin treatment attenuates VEGFC-induced phosphorylation of ERK in endothelial cells. (**A**) Western blot analysis of lysates isolated from human dermal lymphatic microvascular endothelial cells (HMVECs) treated with either 0.05% DMSO, 5 μM sunitinib malate (SM), 3,4-Difluorobenzocurcumin (CDF) at indicated concentrations, or 5 μM curcumin (CM) for 2 h and stimulated with vascular endothelial growth factor C (VEGFC) for 20 min (n ≥ 4). Protein levels of pERK1/2, total ERK1/2, pAKT, total AKT, and Tubulin were assessed. CDF treatment results in a dose-dependent reduction of phosphorylated ERK (pERK) level. The full-length blots are presented in Figure S4. (**B**–**C’**) Lateral confocal images of 32 hpf *Tg(fli1a:nEGFP)* embryos treated with either 0.1% DMSO (**B**) or 2.5 μM CDF (**C**) immunostained with anti-pErk (red) and anti-GFP (green) antibodies. CDF blocks phosphorylation of Erk in venous endothelial cells in vivo. Images (**B’**,**C**); represent the anti-pErk staining of images (**B**,**C**). (**D**) Quantification of pErk and *fli1a:EGFP*-positive nuclei per somite in the posterior cardinal vein (PCV) of 32 hpf *Tg(fli1a:nEGFP)* embryos treated with either 0.1% DMSO (n = 19 embryos) or 2.5 μM CDF (n = 21 embryos). (**E**) Western blot analysis of lysates isolated from 3 dpf zebrafish larvae treated with either 0.1% DMSO, 20 μM SM, 2 μM PD0325901, CDF at indicated concentrations, or 10 μM CM (n = 4). CDF is not a general inhibitor of Erk phosphorylation. Protein levels of pErk1/2, total Erk1/2, and Actin were assessed. The full-length blots are presented in Figure S5. Statistical test: Mann-Whitney test was conducted for graph (**D**). *p* ≤ 0.001 (***). Scale bar: 50 μm.

VEGFC is essential for lymphatic development as deletion of VEGFC results in lack of lymphatic sprouting and migration in both fish and mammals [34,36,37,38,39,40]. VEGFC is able to bind to both VEGFR2 and VEGFR3 to activate downstream serine/threonine kinases such as ERK and AKT in LECs [41]. Hence, CDF may exert its anti-lymphangiogenic effect by targeting VEGFC-induced VEGFR-dependent phosphorylation of endothelial ERK and/or AKT. To investigate this, we used a human endothelial cell culture system with human dermal lymphatic microvascular endothelial cells (HMVECs). As expected, treatment with SM diminished VEGFC-induced ERK and AKT phosphorylation Figure 3A, Figures S3A,B and S4). Treatment of HMVECs with CDF resulted in a dose-dependent reduction of phosphorylated ERK (pERK) level, with inhibition comparable to 5 μM SM observed in HMVECs treated with 1 μM CDF (Figure 3A, Figures S3A and S4). Treatment of HMVECs with CDF did not significantly inhibit phosphorylation of AKT (Figure 3A, Figures S3B and S4). Treatment with curcumin at 5 μM did not reduce VEGFC-induced phosphorylation of ERK and AKT in HMVECs (Figure 3A, Supplementary Figures S3A,B and S4).

To confirm this in vivo, we investigated EC pErk levels in zebrafish. Venous EC (PCV) phosphorylation of Erk during lymphatic sprouting in zebrafish is driven by the Vegfc/Flt4 pathway [42,43]. Consistent with our in vitro data, 32 hpf *fli1a:nEGFP* embryos treated with CDF had a reduced number of pErk-positive ECs in the PCV (Figure 3B–D). In comparison, intersegmental artery (aISV) pErk staining has been previously shown to be Vegfa/Vegfr2 (Kdr/Kdrl)-dependent [44] and the number of pErk-positive ECs in aISVs and the dorsal longitudinal anastomotic vessel (DLAV) was unchanged in 32 hpf *fli1a:nEGFP* embryos treated with CDF (Figure S3C–E). This indicates that CDF at 2.5 μM specifically inhibits Erk activation in venous ECs (VECs) but not in arterial ECs in zebrafish. 

Mitogen-activated protein kinase kinase (MEK) inhibitors are also able to reduce the number of pErk-positive ECs and inhibit lymphatic sprouting in zebrafish [43]. To test if CDF is a MEK inhibitor, we conducted western blot analysis to determine whether CDF treatment reduces whole organism pErk levels in zebrafish. While PD0325901, a selective MEK inhibitor, at 2 μM reduced whole organism pErk levels, CDF at 1 and 2.5 μM, and curcumin at 10 μM failed to show this response (Figure 3E, Figure S3F and S5). This suggests that CDF is not a MEK inhibitor and that the reduction of pERK in ECs is likely due to inhibition of VEGFC-induced vascular signalling. In summary, CDF is able to inhibit VEGFC-dependent phosphorylation of endothelial ERK in vitro and VECs *in vivo.*

Next, we sought to investigate how CDF could be inhibiting the Vegfc/Flt4/Erk pathway. Previous studies had suggested that curcumin is able to reduce mRNA levels of *VEGFR3* in vitro and in vivo [9,10]. However, qPCR analysis for *vegfc* and *flt4* in embryos treated with either DMSO or 2.5 μM CDF revealed no significant changes in mRNA levels (Figure S6A). We next investigated whether CDF inhibits VEGFR3 kinase activity. To this end, we utilized the Z’-LYTE™ kinase assay which tests the efficiency of kinases in phosphorylating synthetic peptide substrates that contain corresponding phosphorylation sites. As expected, SM was able to impair the kinase activity of VEGFR1-3, at a concentration of 1 μM (Figure S6B). However, CDF was not able to inhibit the kinase activity of VEGFR1-3 at 1 μM, and was only able to partially inhibit the kinase activity of VEGFR2 at 5 μM (Figure S6B). This suggests that unlike SM, CDF does not inhibit lymphangiogenesis by blocking the kinase activity of VEGFR3 and may have a distinct mechanism of action.

### 2.4. CDF Rescues Vascular Hyperplasia in a Zebrafish Model of Vegfc-Overexpression

**Figure 4:**
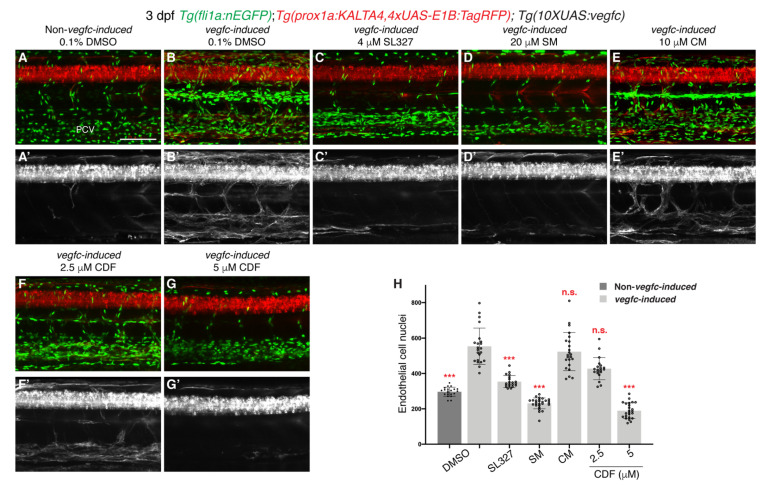
3,4-Difluorobenzocurcumin inhibits pathological phenotypes associated with *vegfc* overexpression. (**A**–**G’**) Lateral confocal images of either a 3 dpf *Tg(prox1a:KALTA4,4xUAS-E1B:TagRFP);Tg(fli1a:nEGFP)* larva (Non*-vegfc-induced*) treated with 0.1% DMSO (**A**,**A’**), or 3 dpf *Tg(prox1a:KALTA4,4xUAS-E1B:TagRFP);Tg(10XUAS:vegfc);Tg(fli1a:nEGFP)* larvae (*vegfc-induced*) treated with either 0.1% DMSO (**B**,**B’**), 4 μM SL327 (**C**,**C’**), 20 μM sunitinib malate (SM, **D**,**D’**), 10 μM curcumin (CM, **E**,**E’**), 2,5 μM 3,4-Difluorobenzocurcumin (CDF, **F**,**F’**), or 5 μM CDF (**G**,**G’**). Pathological vascular phenotypes in *vegfc-induced* embryos are rescued by CDF treatment. Images (**A’**–**G’**) represent the *Tg(prox1a:KALTA4,4xUAS-E1B:TagRFP)* expression of images (**A**–**G**). To avoid the robust *prox1a* expression in muscle cells, (**A’**–**G’**) are maximum projection images of only the z stacks that contain the posterior cardinal vein. Images (**B’**) (21/22 embryos), (**E’**) (23/23 embryos) and (**F’**) (14/20 embryos) show embryos with increased *prox1a:KALTA4,4xUAS-E1B:TagRFP* expression in venous endothelial cells. This pathological phenotype is rescued in images (**C’**) (20/20 embryos), (**D’**) (27/27 embryos) and (**G’**) (21/24 embryos). (**H**) Quantification of *fli1a:EGFP*-positive ECs across 4.5 somites in either 3 dpf *non-vegfc-induced* treated with 0.1% DMSO (n = 21 embryos) or 3 dpf *vegfc-induced* larvae treated with either 0.1% DMSO (n = 22 embryos), 4 μM SL327 (n = 20 embryos), 20 μM SM (n = 27 embryos), 10 μM CM (n = 23 embryos), or CDF at 2.5 μM (n = 20 embryos) or 5 μM (n = 24 embryos). PCV: posterior cardinal vein. Statistical test: Kruskal-Wallis test was conducted for graph H. *p* ≤ 0.001 (***) and n.s. indicates not significant. Scale bar: 100 μm.

VEGFC upregulation contributes to pathological increase in lymphangiogenesis in various diseases [45,46,47,48,49]. Upregulation of *vegfc* in zebrafish results in vastly increased EC proliferation and *prox1a* induction in VECs that is completely dependent on the Vegfc/Flt4 pathway [30]. We therefore tested whether CDF is able to rescue this pathological phenotype. As previously reported [30], 3 dpf *Tg(prox1a:KALTA4,4xUAS-E1B:TagRFP);Tg(10XUAS:vegfc);Tg(fli1a:nEGFP)* compound transgenic line , which overexpresses Vegfc from all *prox1a*-expressing cells (henceforth described as *vegfc-induced*) had increased EC number and a notable upregulation of *prox1a:KALTA4,4xUAS-E1B:TagRFP* expression in VECs when compared to 3 dpf DMSO-treated control siblings (Figure 4A,B,H). As expected, SL327, a selective MEK inhibitor, or SM rescued the increased *prox1a:KALTA4,4xUAS-E1B:TagRFP* expression in VECs and EC proliferation in *vegfc-induced* larvae, while 10 μM curcumin did not (Figure 4C–E’,H). CDF blocked the EC proliferation phenotype and reduced induction of *prox1a:KALTA4,4xUAS-E1B:TagRFP* expression in 3 dpf *vegfc-induced* larvae in a dose-dependent manner (Figure 4F–H). Collectively, these results indicate that CDF may be an effective drug against diseases associated with pathological increase in VEGFC-induced lymphangiogenesis.

### 2.5. Brief Treatment of CDF Displays Prolonged Inhibition of Lymphangiogenesis

**Figure 5:**
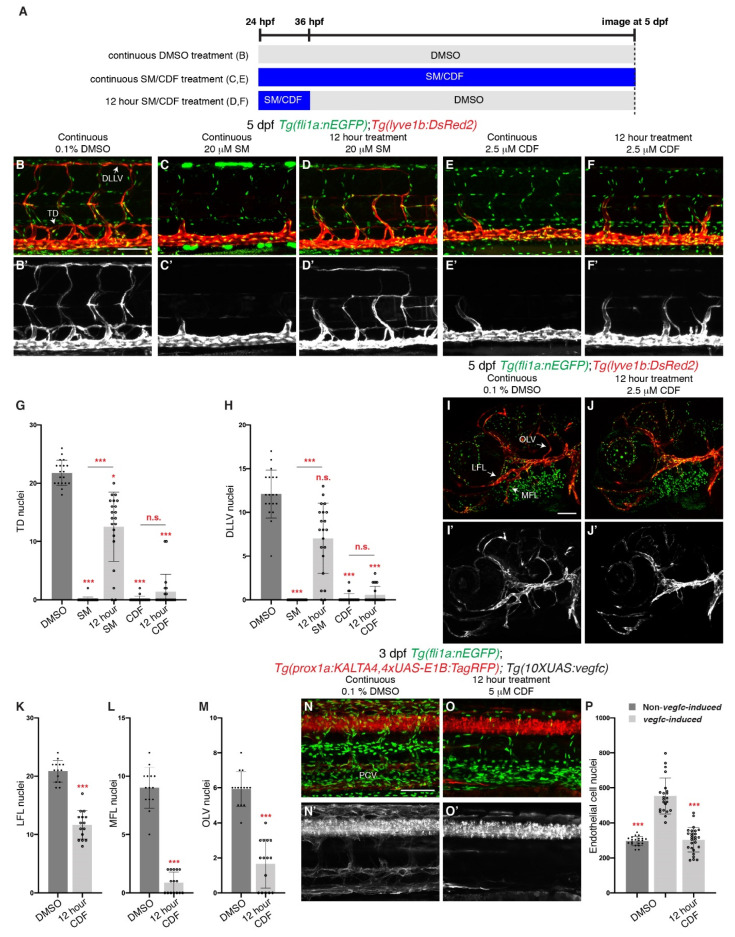
Brief treatment of 3,4-Difluorobenzocurcumin is sufficient to completely inhibit trunk and facial lymphatic development. (**A**) Schematic representation of the treatment schedule for larvae in images (**B**–**F**) and images (**I**,**J**). (**B**–**F’**) Lateral confocal images of 5 dpf *Tg(fli1a:nEGFP);Tg(-5.2lyve1b:DsRed2)* larvae either continuously treated with 0.1% DMSO (**B**,**B’**), 20 μM sunitinib malate (SM, **C**,**C’**) or 2.5 μM 3,4-Difluorobenzocurcumin (CDF, **E**,**E’**), or treated for 12 h with 20 μM SM (**D**,**D’**) or 2.5 μM CDF (**F**,**F’**). 12 h treatment of CDF inhibits trunk lymphatic development. Images (**B’**–**F’**) represent the *Tg(-5.2lyve1b:DsRed2)* expression of images (**B**–**F**). (**G**,**H**) Quantification of thoracic duct (TD, **G**) or dorsal longitudinal lymphatic vessel (DLLV, **H**) nuclei across 9 somites in 5 dpf *Tg(fli1a:nEGFP);Tg(-5.2lyve1b:DsRed2)* larvae treated with either 0.1% DMSO (n = 19 larvae), 20 μM SM (n = 24 larvae) or 2.5 μM CDF (n = 21 larvae), or treated for 12 h with 20 μM SM (n = 21 larvae) or 2.5 μM CDF (n = 21 larvae). (**I**–**J’**) Lateral confocal images of *Tg(fli1a:nEGFP);Tg(-5.2lyve1b:DsRed2)* larvae treated with either 0.1% DMSO (**I**,**I’**) or with 2.5 μM CDF for 12 h, then with 0.1% DMSO up to 5 dpf (**J**,**J’**). 12 h treatment of CDF inhibits facial lymphatic development. Images (**I’**,**J’**) represent the *Tg(-5.2lyve1b:DsRed2)* expression of images (**I**,**J**). (**K**–**M**) Quantification of lateral facial lymphatic (LFL, **K**, n ≥ 14), medial facial lymphatic (MFL, **L**, n ≥ 14), or otolithic lymphatic vessel (OLV, **M**, n ≥ 14) nuclei in 5 dpf *Tg(fli1a:nEGFP);Tg(-5.2lyve1b:DsRed2)* larvae either continuously treated with 0.1% DMSO (n = 14 larvae) or treated for 12 h with 2.5 μM CDF (n = 15 larvae). Datasets for 0.1% DMSO-treated 5 dpf *Tg(fli1a:nEGFP);Tg(-5.2lyve1b:DsRed2)* larvae are taken from Figure 1P–R. (**N**–**O’**) Lateral confocal images of 3 dpf *Tg(prox1a:KALTA4,4xUAS-E1B:TagRFP);Tg(10XUAS:vegfc);Tg(fli1a:nEGFP)* larvae (*vegfc-induced*) treated with either 0.1% DMSO (**N**,**N’**) or with 5 μM CDF for 12 h, then with 0.1% DMSO up to 3 dpf (**O**,**O’**). Pathological vascular phenotypes in *vegfc-induced* embryos are rescued by 12 h treatment of CDF. Images (**N’**,**O’**) represent the *Tg(prox1a:KALTA4,4xUAS-E1B:TagRFP)* expression of images (**N**,**O**). To avoid the robust *prox1a* expression in muscle cells, (**N’**,**O’**) are maximum projection images of only the z stacks that contain the posterior cardinal vein. Image (**N’**) (21/22 embryos) shows an embryo with increased *prox1a:KALTA4,4xUAS-E1B:TagRFP* expression in venous endothelial cells. This pathological phenotype is rescued in image (**O’**) (24/27 embryos). (**P**) Quantification of *fli1a:EGFP*-positive ECs across 4.5 somites in either 3 dpf Non*-vegfc-induced* (n = 21 embryos) or 3 dpf *vegfc-induced* larvae treated with either 0.1% DMSO (n = 22 embryos), or for 12 h with 5 μM CDF (n = 27 embryos). Datasets for 0.1% DMSO-treated 3 dpf Non*-vegfc induced* and *vegfc-induced* larvae are taken from Figure 4H. PCV: posterior cardinal vein. Statistical test: Mann-Whitney test were conducted for graph (**K**–**M**). Kruskal-Wallis test were conducted for graphs (**G**,**H**,**P**). *p* ≤ 0.001 (***) and n.s. indicates not significant. Scale bars: 100 μm.

Some tyrosine kinase inhibitors demonstrate the ability to inhibit their molecular target for a longer duration, allowing infrequent administration of the drug to confer long-lasting effects [50,51]. When SM is treated for 12 h and subsequently washed, lymphatic development was not completely inhibited, with significantly higher TD and DLLV LEC numbers when compared to larvae continuously treated with SM (Figure 5A–D’,G,H). We repeated the experiment using CDF and found that unlike SM, 12 h treatment of CDF severely reduced TD and DLLV LEC numbers, comparable to larvae treated continuously, at 5 dpf when compared to DMSO-treated larvae (Figure 5E–H). In addition, 12 h treatment was sufficient to inhibit facial lymphatic development (Figure 5I–M). Although minor craniofacial defects were present, the major cartilages formed normally and the general phenotype of these larvae was similar to DMSO treated larvae, with significantly reduced pericardial oedema formation and normal inflation of the swim bladder (Figure S1). As 12 h treatment with CDF was sufficient to inhibit lymphangiogenesis, we also investigated whether 12 h treatment inhibits the pathological hyperplasia phenotype in our *vegfc-induced* larvae. Indeed, 12 h treatment of 5 μM CDF reduced EC proliferation and *prox1a:KALTA4,4xUAS-E1B:TagRFP* expression in the VECs in 3 dpf *vegfc-induced* larvae (Figure 5N–P). Together, these results suggest that the efficacy of CDF may be distinct from those of known VEGFR kinase inhibitors such as SM.

## 3. Discussion

Inhibiting aberrant lymphatic growth in models of human diseases such as cancer, lymphatic malformation, organ graft rejection, dry eye disease and allergic eye disease, has been shown to alleviate their pathological symptoms (reviewed in [3]), highlighting the need for anti-lymphangiogenic agents. Here, as part of a larger biodiscovery platform assessing poorly studied candidate small molecules, we focused on CDF which has been implicated in cancer [14,15,16,17,18,19] but not in lymphangiogenesis. We identified CDF as a novel inhibitor of lymphangiogenesis using both zebrafish and HMVECs. Mechanistically, CDF is able to inhibit VEGFC-induced phosphorylation of ERK in vitro in human cells, and in venous endothelial cells in zebrafish. Furthermore, CDF inhibits both initial sprouting of LECs and their ongoing development, as shown with carefully staged treatments at different stages of development. These observations together demonstrate that CDF blocks the same stages of lymphatic development controlled by VEGFC-VEGFR3 signalling. Consistent with acting via inhibition in the Vegfc/Flt4/Erk pathway, CDF treatment attenuated increased EC proliferation and increased *prox1a* expression driven by the direct transgenic overexpression of *vegfc*.

Despite the high similarity in chemical structures between CDF and the parental compound curcumin [14], we saw no evidence for any anti-lymphangiogenic activity of curcumin even at high doses. Wang and colleagues reported that curcumin is able to reduce the VEGFC-induced increase in LYVE1-positive cells in implanted matrigels in mice using a flow cytometry analysis [9]. Similarly, Da and colleagues showed that curcumin treatment reduces the density of intratumoral LYVE1-positive vessels in mice xenotransplanted with gastric cancer cells [10]. However, LYVE1 staining alone is not sufficient to differentiate lymphatic vessels from other cells, such as macrophages, which also express LYVE1 [52,53]. Hence, the anti-lymphangiogenic activity of curcumin may be minimal in these studies and while we cannot exclude that curcumin may be anti-lymphangiogenic at very high concentrations, we were unable to investigate this due to general toxicity at such concentrations.

Of note, similar to *vegfc* or *flt4* mutants, treatment with 2.5 μM CDF did not completely inhibit craniofacial (eg. LFL) lymphatic development but did inhibit trunk lymphangiogenesis [37,39,43]. CDF did not reduce *vegfc* or *flt4* mRNA levels, and so CDF may inhibit Vegfc maturation, Vegfc/Flt4 binding, or downstream signalling driven at the level of the receptor. It is unlikely to inhibit more general MAPK signalling downstream of the ligand/receptor pairing because it does not inhibit ERK signalling in the embryo overall. Our kinase assay showed that CDF partially inhibits VEGFR2 kinase activity but not VEGFR3 activity. The ability of CDF to partially inhibit VEGFR2 kinase activity was not surprising as CDF at a higher concentration (10 μM) inhibits ISV formation in zebrafish. However, the overall inhibition of VEGFR2 kinase activity was less than 50% and so it is difficult to appreciate why this would lead to a loss of lymphatics. In addition, co-repression of Vegfr2 (Kdr) and Flt4 activity should result in complete inhibition of facial lymphatic development [54]. Further mechanistic studies are clearly needed to uncover the precise target (or targets) of CDF activity in lymphangiogenesis. Despite this, just 12 h treatment with CDF resulted in prolonged inhibition of lymphangiogenesis via continued inhibition of the Vegfc/Flt4/Erk pathway compared with the FDA approved standard of care molecule in renal cell carcinoma and imatinib-resistant gastrointestinal stromal tumour, SM. This could suggest that CDF has an alternative and unique target compared with SM, or CDF may have unique pharmacodynamics and thus be capable of prolonged inhibition of known targets. Either way, these observations suggest that infrequent treatment with CDF may be sufficient to confer strong anti-lymphangiogenic effect. CDF is actively being investigated as an anti-cancer drug candidate and based on our findings it would seem important for these studies to assess its anti-tumour-associated lymphangiogenic and anti-metastatic activity.

Overall, our study demonstrates the ability to characterise promising anti-lymphangiogenic drugs using zebrafish. This utility of the zebrafish model is due to the high level of evolutionary conservation of key molecular pathways that control lymphangiogenesis [20,54]. Thus, this serves as a strong proof-of-principle, justifying increased drug screening efforts focussed upon zebrafish lymphatics, with future efforts potentially identifying both anti-lymphangiogenic and pro-lymphangiogenic compounds. It will be of great interest to understand if CDF as a therapeutic drug may also have applications more broadly in diseases associated with lymphangiogenesis such as lymphatic malformation, organ transplant rejection or cardiovascular diseases.

## 4. Materials and Methods

### 4.1. Zebrafish Maintenance

Zebrafish transgenic lines used were *Tg(fli1a:EGFP)^y1^* [55], *Tg(fli1a:nEGFP)^y7^* [56], *Tg(-5.2lyve1b:DsRed2)^nz101^*, *Tg(-5.2lyve1b:EGFP)^nz150^* [26], *TgBAC(prox1a:KALTA4,4xUAS-E1B:TagRFP)^nim5^* [57], and *Tg(10XUAS:vegfc)^uq2bh^* [30]. Larvae/embryos were anaesthetised in 0.08 mg/mL tricaine and imaged as previously described [31].

### 4.2. Chemical Administration

16 hpf (for angiogenic quantification) or 24 hpf (for lymphatic quantification) embryos were treated with either vehicle control (0.1% DMSO), 20 μM SM (LC laboratories, MA, USA), 4 μM SL327 (Merck, NJ, USA), 2 μM PD0325901 (Selleckchem, TX, USA), 10 μM curcumin (Acros Organics, NJ, USA), or CDF (LKT laboratories Inc, MN, USA) in E3 medium at indicated concentrations. To wash out CDF or SM at 12 h post-treatment (36 hpf), treatment solution, which contained either 0.1% DMSO, 20 μM SM, or CDF at indicated concentrations in E3 medium, was replaced with 0.1% DMSO in E3 medium and subsequently washed with 0.1% DMSO in E3 medium 3 times. These embryos were then treated with 0.1% DMSO in E3 medium. For delayed treatment of CDF, 24 hpf embryos were treated with 0.1% DMSO in E3 medium until 2.5 dpf, then the treatment solution was replaced with 2.5 μM CDF in E3 medium. All embryos were co-treated with 0.003% (*w*/*v*) 1-phenyl-2-thiourea in E3 medium to stall pigmentation.

### 4.3. Quantification of Angiogenesis and Lymphangiogenesis in Zebrafish

Fully formed ISVs were manually quantified between 6th to the 20th somite in 48 hpf *Tg(fli1a:EGFP)* embryos. TD tissue fragments in equivalent regions were manually quantified in 6 dpf *Tg(lyve1b:DsRed2)* larvae. Manual quantification was performed under an Olympus IX81 fluorescence microscope. To quantify TD and DLLV LEC number and PL number, the trunk region of *Tg(lyve1b:DsRed2);Tg(fli1a:nEGFP)* larvae and embryos was imaged at the indicated stages using a LSM 710 FCS inverted confocal microscope and quantified as previously described [31]. Secondary sprout number in 36 hpf *Tg(lyve1b:EGFP)* embryos were quantified as previously described [26]. To quantify the LFL, MFL and the OLV of the facial lymphatic network, images of the facial region of 5 dpf *Tg(lyve1b:DsRed2);Tg(fli1a:nEGFP)* larvae were taken using the LSM 710 FCS inverted confocal microscope. The number of LFL, MFL, and OLV LECs within the regions shown in Figure S7 were then quantified manually using the cell counter function in the Fiji image processing software (version 1) [58]. Images of 3dpf *Tg(prox1a:KALTA4,4xUAS-E1B:TagRFP);Tg(10XUAS:vegfc);Tg(fli1a:nEGFP)* larvae were taken using the LSM 710 FCS inverted confocal microscope. The *fli1a:nEGFP*-positive EC number was then quantified as previously described using Imaris x64 software (Bitplane, Belfast, UK, version 9.5.1) [30]. Only embryos/larvae with blood flow were imaged and quantified.

### 4.4. Alcian Blue Staining

Alcian Blue staining was done as described previously [59] with modifications. Briefly, 5 dpf larvae treated with either 0.1% DMSO, 2.5 µM CDF, or 12 h with 2.5 µM CDF were fixed overnight in 4% paraformaldehyde at 4 °C. Fixed larvae were washed with PBS-Tween 20 (0.1%) at room temperature, and sequentially dehydrated in ethanol. After, larvae were incubated in Alcian blue solution (0.2 mg/mL Alcian Blue in 70% ethanol and 30% glacial acetic acid, filtered through 0.2 µm syringe filter before use) at room temperature overnight. Larvae were rehydrated, bleached, and imaged as previously described [59].

### 4.5. Cell Culture

HMVECs (Lonza, Basel, Switzerland) were maintained from passage 3 to 7 in EGM-2MV media per supplier’s instructions. Cells were seeded at a density of 2.85 × 10^5^ cells/well in 6-well plate, grown to 80% confluence, and serum-starved overnight. After, cells were treated with either 0.05% DMSO, 5 µM SM, 5 µM curcumin or CDF at indicated concentrations for 2 h. Treated cells were then stimulated with human recombinant VEGFC (500 ng/mL, R&D Systems, MN, USA) for 20 min before collection for analysis.

### 4.6. Western Blot and qPCR Analysis

To isolate zebrafish protein, 3 dpf zebrafish larvae treated with either 0.1% DMSO, 2 µM PD0325901, 10 µM curcumin or CDF at indicated concentrations were deyolked before being lysed in radioimmunoprecipitation assay (RIPA) buffer containing 1x protease and phosphatase inhibitor cocktail (Thermo Fisher Scientific, MA, USA). Cell lysates were isolated as previously described [60]. Western blot analysis was performed as previously described [60]. Primary antibodies used for western blot analysis were ERK1/2 (#9102), pERK (#9101), AKT (#2938), pAKT (#4060), β-Tubulin (#2128) (Cell Signaling Technology, MA, USA), and Actin antibodies (#mab1501, Merck Millipore, MA, USA). Zebrafish mRNA was collected from 36 hpf zebrafish larvae treated with either 0.1% DMSO or 2.5 µM CDF as previously described [39]. qPCR was performed as previously described [39] and primer details can be found in Table S1.

### 4.7. Immunofluorescence Staining of Phosphorylated Erk in Zebrafish

Primary antibodies used for pErk immunofluorescence staining were pERK1/2 (#4370, Cell Signaling Technology, MA, USA) and GFP antibodies (#ab13970, Abcam, Cambridge, UK). Immunofluorescence staining of pErk were done as previously described [31] but with the following modifications. 32 hpf *Tg(fli1a:nEGFP)* embryos treated with either 0.1% DMSO or 2.5 µM CDF were fixed in 4% paraformaldehyde overnight at 4 °C. Fixed embryos were then washed three times with ice cold 100% methanol for 5 min, placed in 3% H_2_O_2_ diluted in 100% methanol for 1 h on ice, then washed three times with ice cold 100% methanol for 5 min and stored at −20 °C for at least two days. After, embryos were washed three times with PBS-Tween 20 (0.1%) at room temperature for 10 min, then embryos were cryoprotected by incubating in 30% sucrose in PBS-Tween 20 (0.1%) overnight at 4 °C. Next, embryos were washed three times with PBS-Tween (0.1%) at room temperature for 10 min and subsequent staining steps were as previously described [31]. Immunostained embryos were imaged using a LSM 710 FCS inverted confocal microscope and pErk and fli1a:nEGFP double positive endothelial nuclei in the PCV, aISVs (only the aISVs on one side of the embryo were quantified) or the DLAV were manually quantified using the cell counter function in the Fiji image processing software as previously described [31,58].

### 4.8. Kinase Assay

The Z’-LYTE™ kinase assay was performed by a service provider (SelectScreen^®^ Kinase Profiling, ThermoFisher Scientific, MA, USA) according to the manufacturer’s protocol. CDF at 1 and 5 μM, and SM at 5 μM were tested for its % kinase activity inhibition for VEGFR1-3.

### 4.9. Statistical Analysis

All statistical analysis was performed using Prism software (GraphPad Prism, Prism 8, version 8.3.0). Analysis was done using either Mann-Whitney test for comparison of two means, or Kruskal-Wallis test for comparison of multiple means. Stars indicate p-value as level of significance with *p* ≤ 0.001 (***), *p* ≤ 0.01 (**), *p* ≤ 0.05 (*), and *p* > 0.05 (not significant, n.s.). Error bars in all graphs represent standard deviation.

## Data Availability

Data is contained within the article and Supplementary Material.

## Supplementary Materials

The following are available online at https://www.mdpi.com/article/10.3390/ph14070614/s1, Figure S1: Brief treatment of 3,4-Difluorobenzocurcumin causes less morphological phenotypes than longer treatment. Figure S2: 3,4-Difluorobenzocurcumin treatment inhibits lymphatic and venous sprouting. Figure S3: 3,4-Difluorobenzocurcumin treatment reduces protein level of pERK in human dermal lymphatic microvascular endothelial cells but does not reduce the whole-organism protein level of pErk in zebrafish. Figure S4: Original western blot images of Figure 2A. Figure S5: Original western blot images of Figure 2E. Figure S6: 3,4-Difluorobenzocurcumin treatment does not reduce *vegfc* and *flt4* mRNA levels, and VEGFR3 kinase activity. Figure S7: Schematic representation of the regions where facial lymphatic vessels were quantified. Table S1: Primer sequences used in this study.
