# Supplementary material for: 3,4-Difluorobenzocurcumin Inhibits Vegfc-Vegfr3-Erk Signalling to Block Developmental Lymphangiogenesis in Zebrafish
Source: Pharmaceuticals (Basel). 2021 Jun 26;14(7):614. doi: 10.3390/ph14070614 (PMC8308560; doi:10.3390/ph14070614)
Supplement: Supplementary file 1 [file pharmaceuticals-14-00614-s001.zip › pharmaceuticals-1242826-supplementary.pdf]

3,4-Difluorobenzocurcumin inhibits Vegfc-Vegfr3-Erk signalling to block developmental lymphangiogenesis in zebrafish

Kazuhide S. Okuda[1,2,3,4,5,#], Mei fong Ng[5], Nur Faizah Ruslan[5], Neil I. Bower[4], Dedrick Soon Seng Song[5], Huijun Chen[4], Sungmin Baek[4,6], Philip S. Crosier[7], Katarzyna Koltowska[4,8], Jonathan W. Astin[7], Pei Jean Tan[5], Benjamin M. Hogan[1,3,4+], Vyomesh Patel[5,+]

[1]Organogenesis *and Cancer Program, Peter MacCallum Cancer Centre, Melbourne, VIC 3000, Australia*

[2]*Sir Peter MacCallum Department of Oncology, University of Melbourne, Melbourne, VIC 3000, Australia*

[3]*Department of Anatomy and Physiology, University of Melbourne, Melbourne, VIC 3000, Australia*

[4]*Division of Genomics of Development and Disease, Institute for Molecular Bioscience, The University of Queensland, St Lucia, QLD 4072, Australia*

[5]*Cancer Research Malaysia, 47500 Subang Jaya, Selangor, Malaysia*

[6]*Stowers Institute for Medical Research, Kansas City, MO 64110, USA*

[7]*Department of Molecular Medicine & Pathology, School of Medical Sciences, The University of Auckland, Auckland 1010, New Zealand*

[8]*Department of Immunology, Genetics and Pathology, Uppsala University, Hammarskjölds väg 20, 751 85, Uppsala, Sweden*

[+]*The last two authors contributed equally to the study*

Author for Correspondence:

[#] Dr Kazuhide Shaun Okuda

E-mail: kazuhideshaun.okuda@petermac.org

Key words: 3,4-Difluorobenzocurcumin, zebrafish, lymphatic, Vegfc, Vegfr3, Erk

Supplementary figures

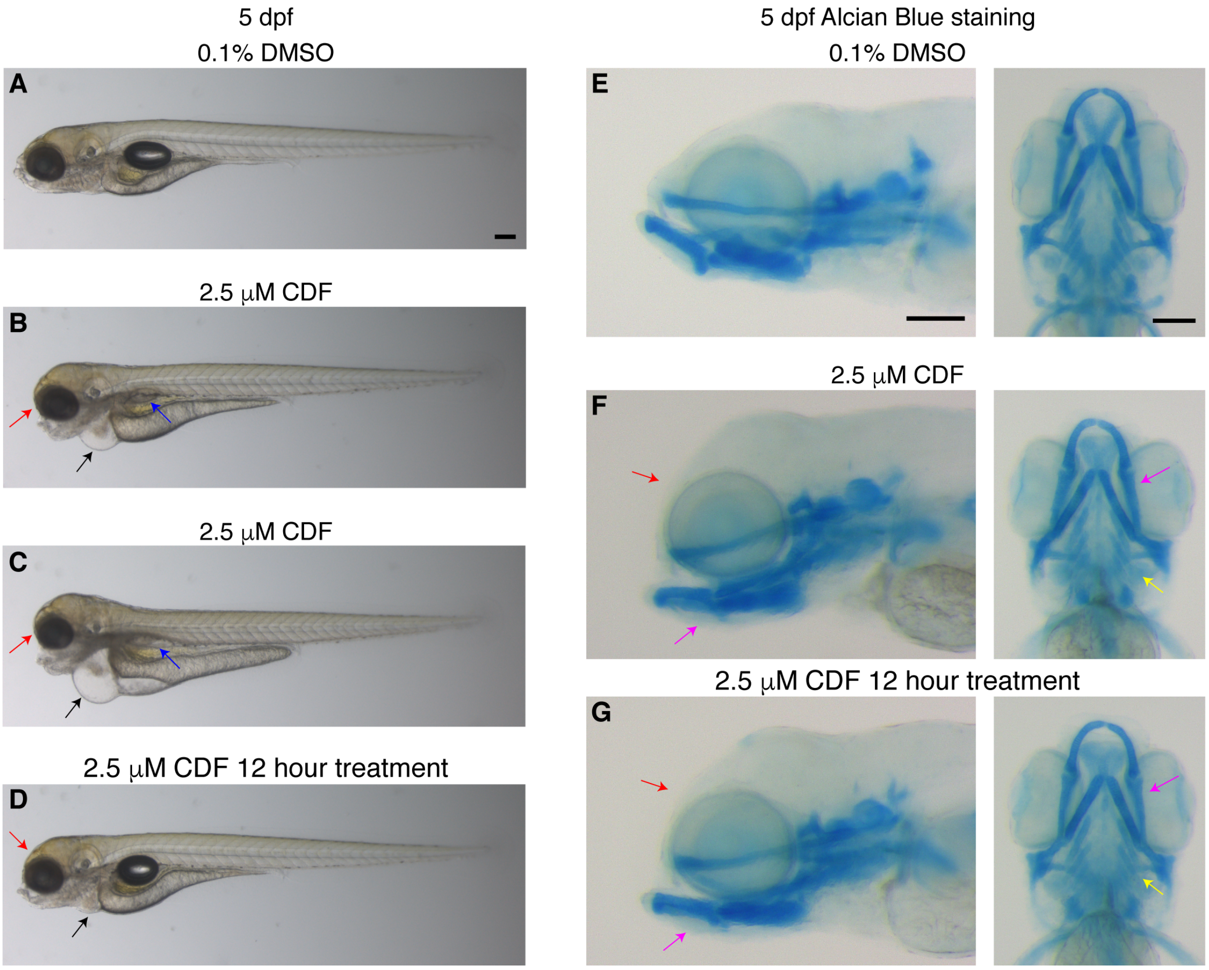

Figure S1: Brief treatment of 3,4-Difluorobenzocurcumin causes less morphological phenotypes than longer treatment.

(A-D) Lateral trans-light images of 5 dpf larvae either continuously treated with 0.1% DMSO (A: 40/40), 2.5 μM 3,4-Difluorobenzocurcumin (CDF, B: 15/40, C: 25/40), or treated for 12 hours with 2.5 μM CDF (D: 34/40). (**E-G**) Lateral (left) and ventral (right) trans-light images of 5 dpf larvae either continuously treated with 0,1% DMSO (E: 45/45), 2,5 μM CDF (F: 41/45), or treated for 12 hours with 2.5 μM CDF (G: 37/45) stained with Alcian Blue. Purple arrows show the major jaw elements that form normally and along which facial lymphatics normally develop. Other arrows show morphological phenotypes (impaired anterior cranial protrusion (red), pericardial oedema (black), lack of swim bladder inflation (blue), delayed pharyngeal

cartilage development (yellow)) associated with treatment with CDF. Brief treatment of CDF causes less morphological phenotypes.

Scale bars: 100 μm.

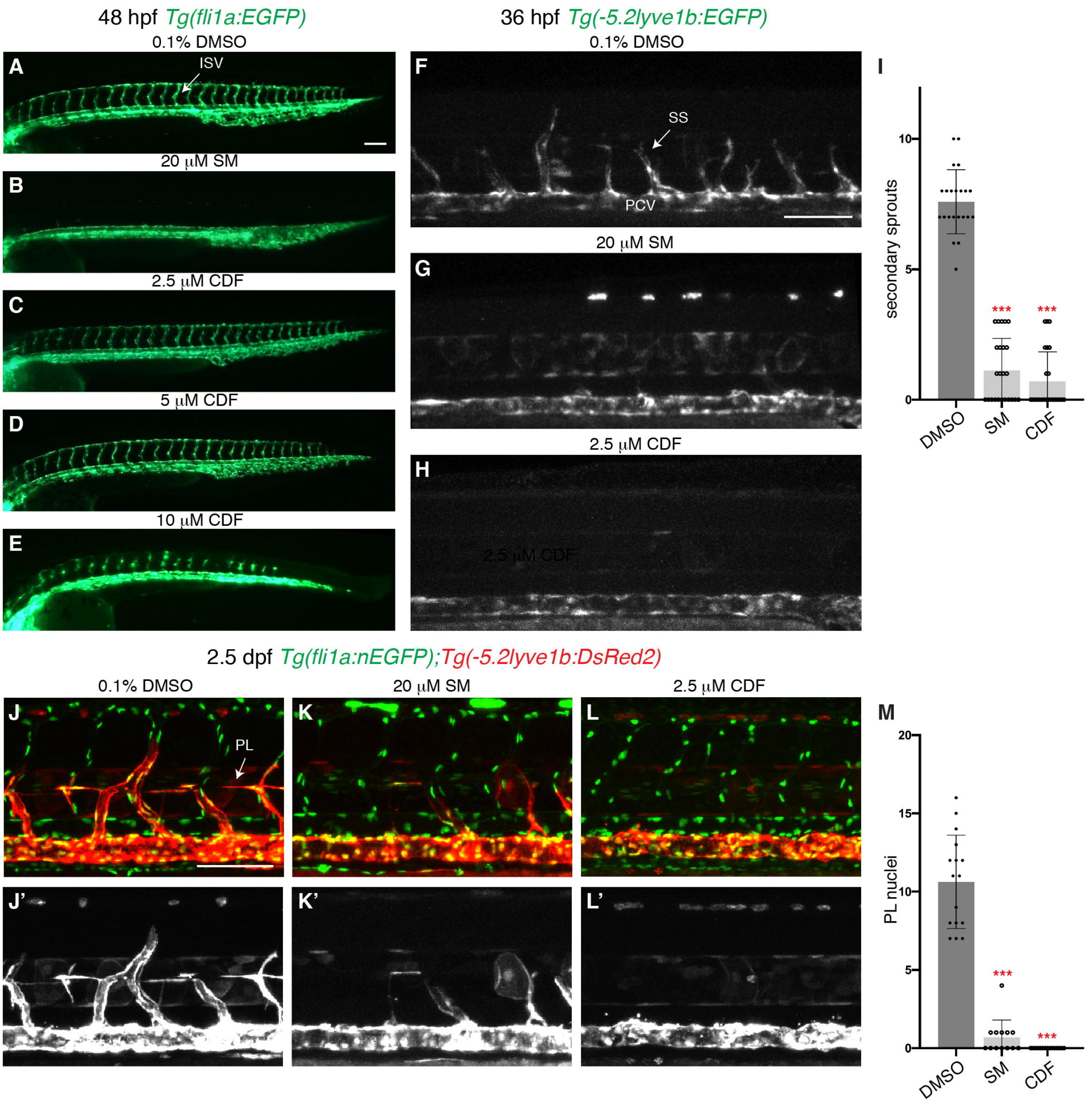

Figure S2: 3,4-Difluorobenzocurcumin treatment inhibits lymphatic and venous sprouting.

(A-E) Lateral fluorescent images of 48 hpf *Tg(fli1a:EGFP)* embryos treated with either 0.1% DMSO (A), 20 μM sunitinib malate (SM, B), or 3,4-Difluorobenzocurcumin (CDF, C-E) at indicated concentrations. CDF at 5 and 2.5 μM do not inhibit primary angiogenesis.

(F-H) Lateral confocal images of 36 hpf *Tg(-5.2lyve1b:EGFP)* embryos treated with either 0.1% DMSO (F), 20 μM SM (G), or 2.5 μM CDF (H). CDF at 2.5 μM blocks secondary sprouting.

(I) Quantification of secondary sprout (SS) number across 10 somites in 36 hpf *Tg(-5.2lyve1b:EGFP)* embryos treated with either 0.1% DMSO (n=22 embryos), 20 μM SM (n=24 embryos), or 2.5 μM CDF (n=24 embryos).

(J-L') Lateral confocal images of 2.5 dpf *Tg(fli1a:nEGFP);Tg(-5.2lyve1b:DsRed2)* embryos treated with either 0.1% DMSO (J,J'), 20 μM SM (K,K') or 2.5 μM CDF (L,L'). Images J'-L' represent the *Tg(-5.2lyve1b:DsRed2)* expression of images J-L. CDF at 2.5 μM inhibits parachordal LEC (PL) formation.

(M) Quantification of PL nuclei across 10 somites in 2.5 dpf *Tg(fli1a:nEGFP);Tg(-5.2lyve1b:DsRed2)* embryos treated with either 0.1% DMSO (n=16 embryos), 10 μM SM (n=13 embryos), or 2.5 μM CDF (n=15 embryos).

ISV: intersegmental vessel, PCV: posterior cardinal vein. Statistical test: Kruskal-Wallis test was conducted for graphs I and M. $p \leq 0.001$ (***). Scale bars: 100 μm.

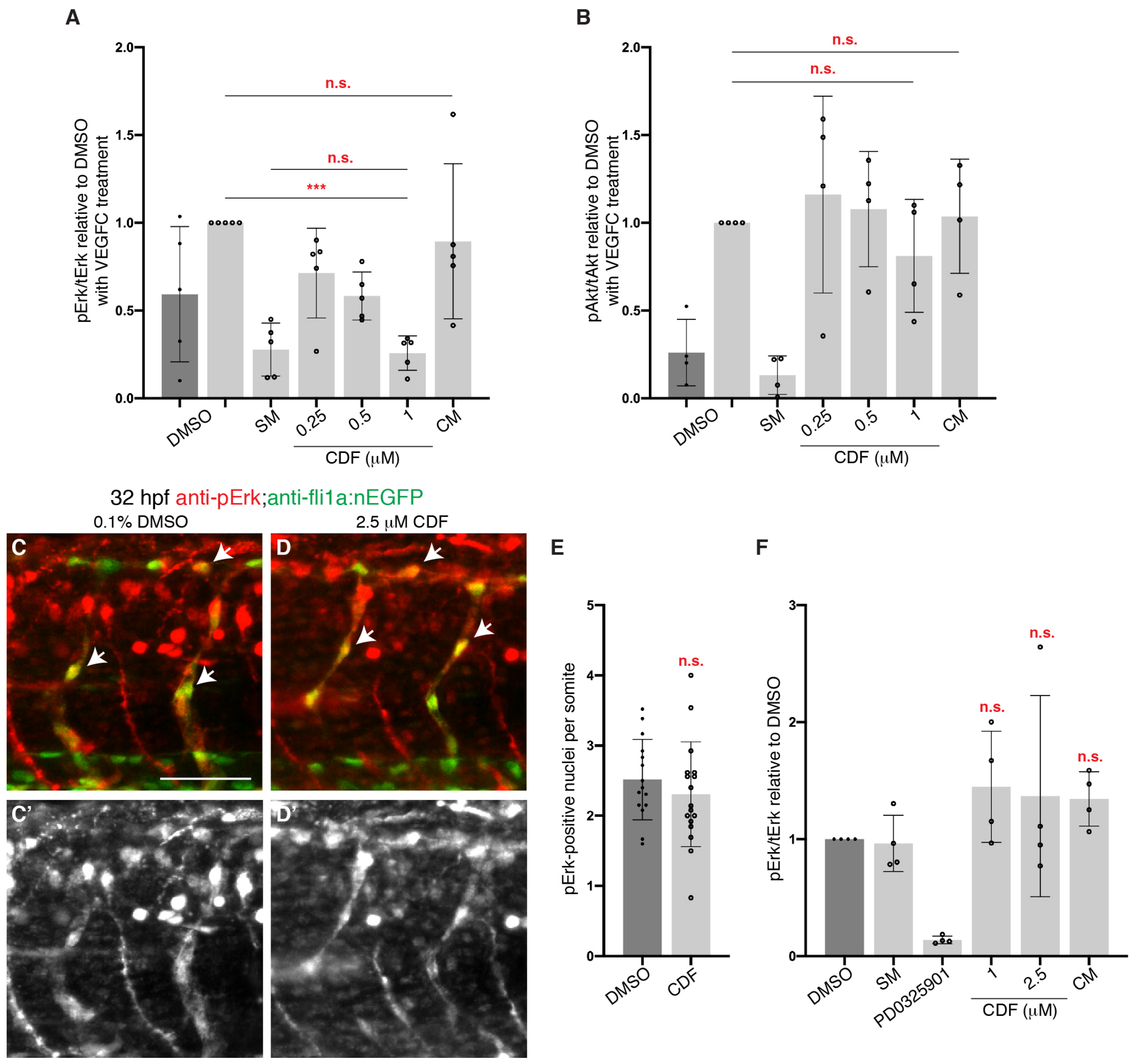

Figure S3: 3,4-Difluorobenzocurcumin treatment reduces protein level of pERK in human dermal lymphatic microvascular endothelial cells but does not reduce the whole-organism protein level of pErk in zebrafish.

(**A,B**) Densitometry analysis of pERK (A) and pAKT (B) protein levels from western blot analysis in **Figure 3A** (n=5 for pERK analysis and n=4 for pAKT analysis). CDF dose-dependently blocks VEGFC-induced ERK phosphorylation in human dermal lymphatic microvascular endothelial cells. pERK and pAKT protein levels were normalized to total ERK (tERK) and tAKT protein levels.

(C-D') Lateral confocal images of 32 hpf *Tg(fli1a:nEGFP)* embryos treated with either 0.1% DMSO (C,C') or 2.5 μM 3,4-Difluorobenzocurcumin (CDF, D,D') immunostained with anti-pErk (red) and anti-GFP (green) antibodies. CDF at 2.5 μM

does not inhibit Erk phosphorylation in arterial endothelial cells. Images C’ and D’ represent the anti-pErk staining of images C and D. White arrows indicate pERK-positive arterial intersegmental vessel (aISV) or dorsal longitudinal anastomotic vessel (DLAV) endothelial cells.

(E) Quantification of pErk and fli1a:EGFP-positive nuclei in aISVs and the DLAV of 32 hpf *Tg(fli1a:nEGFP)* embryos treated with either 0.1% DMSO (n=19 embryos) or 2.5 μM CDF (n=21 embryos).

(F) Densitometry analysis of pErk levels from western blot analysis in **Figure 3E** (n=4). pErk protein levels were normalized to total Erk (tErk) protein levels. CDF at 2.5 μM does not block whole-organism Erk phosphorylation.

Statistical test: Mann-Whitney test was conducted for graph E. Kruskal-Wallis test was conducted for graphs A, B, and F. $p \leq 0.001$ (***) and n.s. indicates not significant. Scale bar: 50 μm.

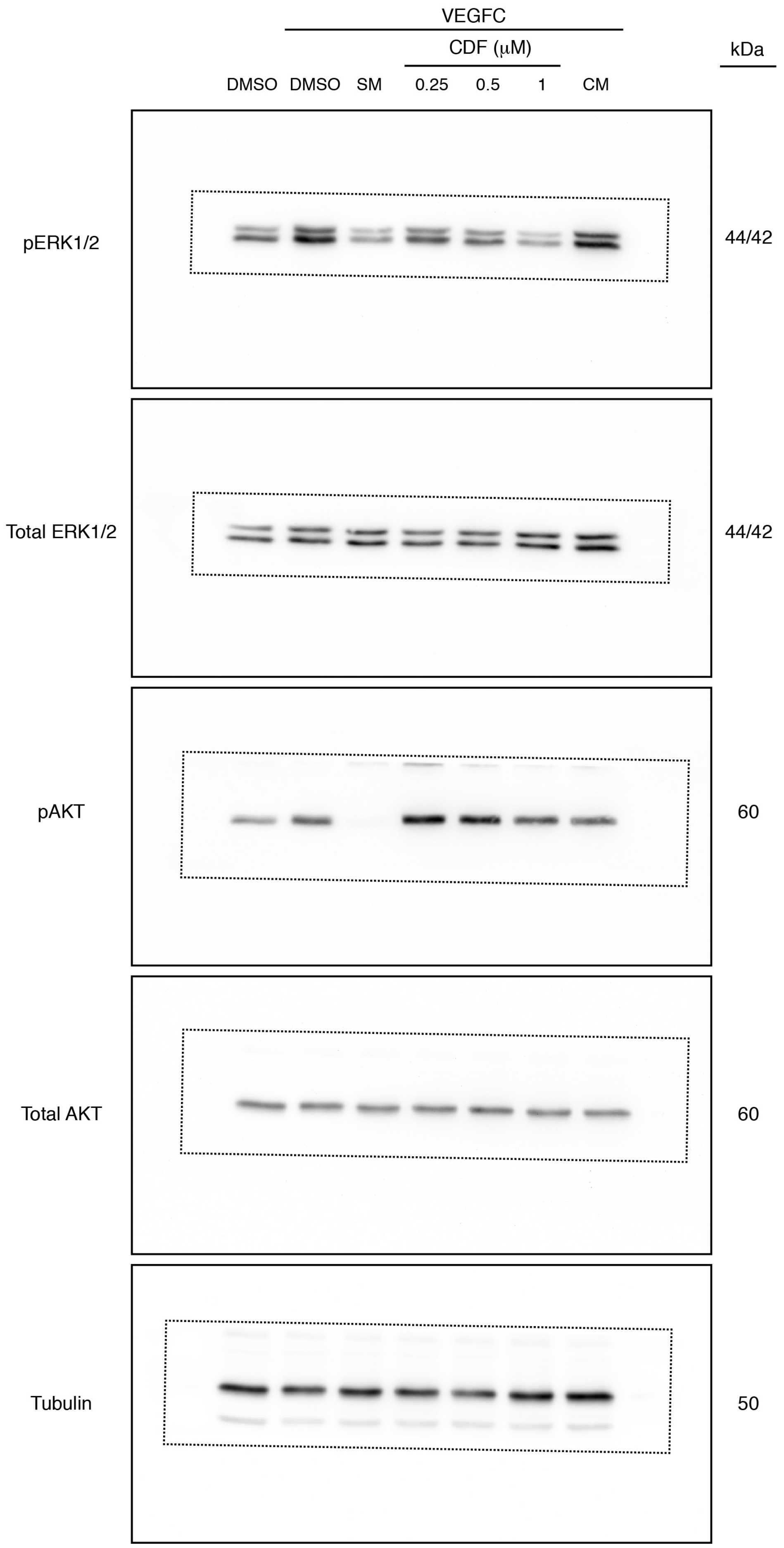
VEGFC
CDF (μM)
kDa
DMSO DMSO SM 0.25 0.5 1 CM
pERK1/2
44/42
Total ERK1/2
44/42
pAKT
60
Total AKT
60
Tubulin
50

Figure S4: Original western blot images of Figure 3A.

Blots were pre-cropped at the dashed lines as indicated before incubation with respective primary antibodies. Images were taken using the ChemiImager^{TM} Imaging System (Alpha Innotech, CA, USA). Original western blot images were processed using the Fiji image processing software.

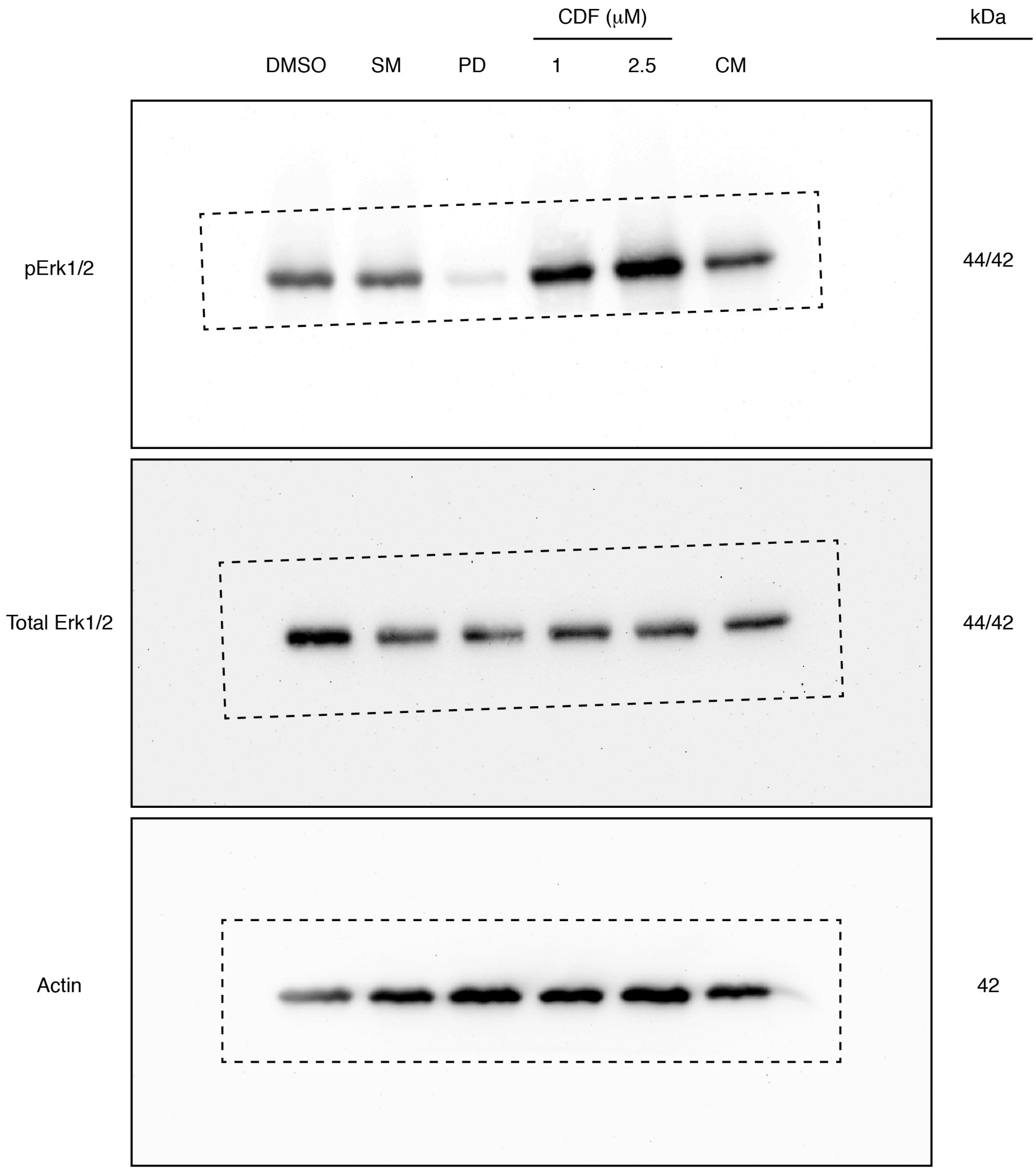

Figure S5: Original western blot images of Figure 3E.

Blots were pre-cropped at the dashed lines as indicated before incubation with respective primary antibodies. Images were taken using the ChemiImager™ Imaging System (Alpha Innotech, CA, USA). Original western blot images were processed using the Fiji image processing software.

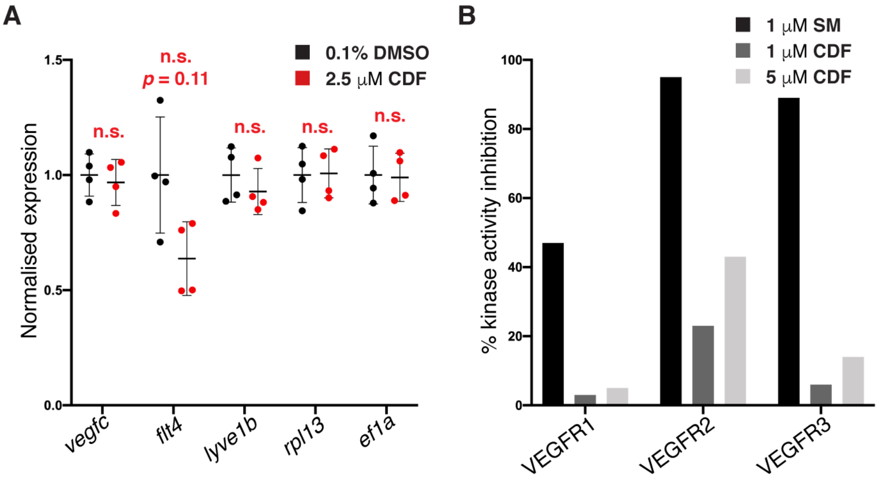

Figure S6: 3,4-Difluorobenzocurcumin treatment does not reduce *vegfc* and *flt4* mRNA levels, and VEGFR3 kinase activity.

(**A**) qPCR analysis of *vegfc*, *flt4*, *lyve1b*, *rpl13*, and *ef1a* mRNA levels in 36 hpf embryos treated with either 0.1% DMSO or 2.5 μM 3,4-Difluorobenzocurcumin (CDF, n=4). CDF at 2.5 μM does not significantly inhibit *vegfc* or *flt4* mRNA levels. (**B**) Quantification of % kinase activity inhibition of either 1 μM sunitinib malate (SM), 1 μM CDF, or 5 μM CDF against VEGFR1 (1 μM SM=47%, 1 μM CDF=3%, 5 μM CDF=5%), VEGFR2 (1 μM SM=95%, 1 μM CDF=23%, 5 μM CDF=43%) and VEGFR3 (1 μM SM=89%, 1 μM CDF=6%, 5 μM CDF=14%) (n=2). CDF is not an inhibitor of VEGFR3 kinase activity at 1 or 5 μM.

Statistical test: Mann-Whitney test was conducted for individual genes in graph A. n.s. indicates not significant.

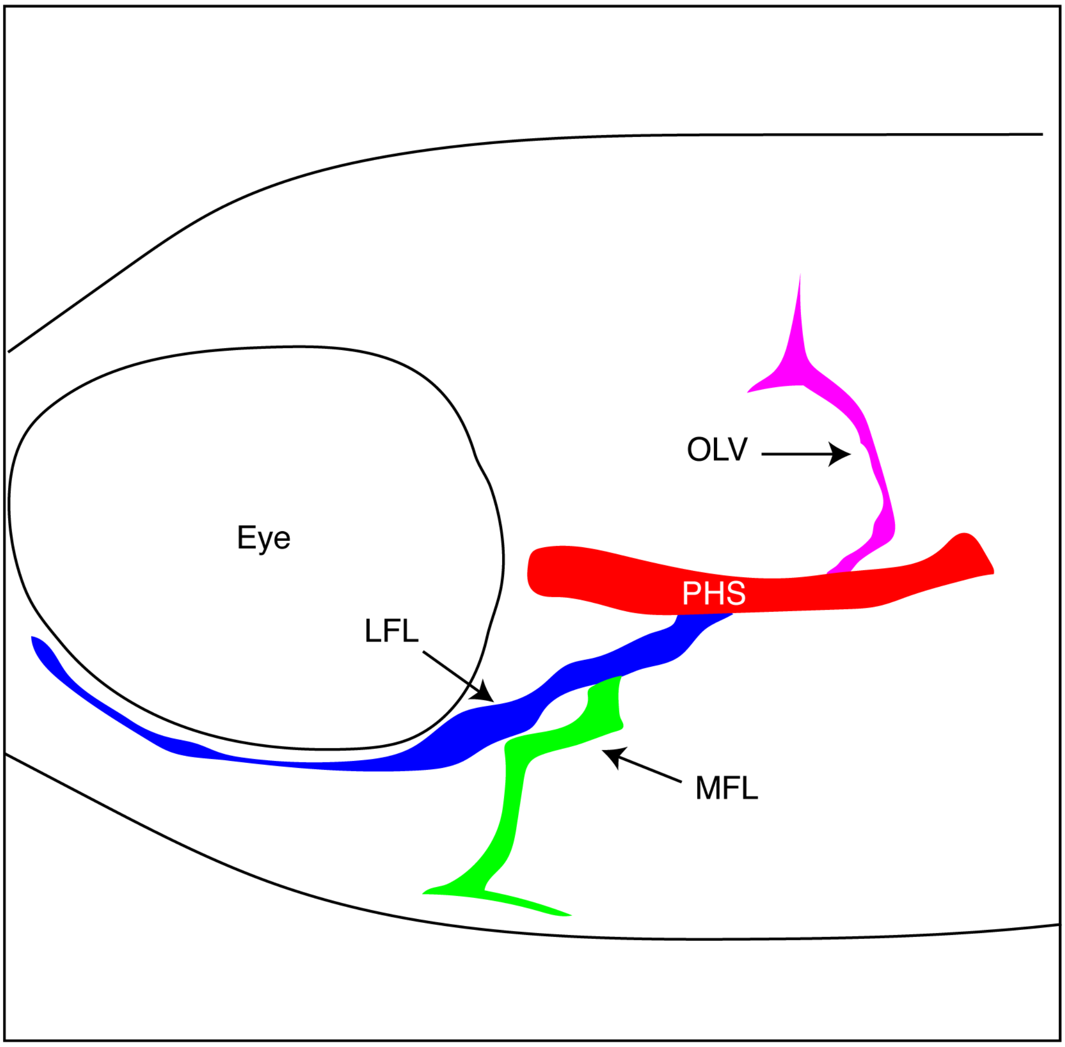

Figure S7: Schematic representation of the regions where facial lymphatic vessels were quantified.

Schematic representation of the regions where lateral facial lymphatic (LFL, blue), medial facial lymphatic (MFL, green), or otolithic lymphatic vessel (OLV, purple) were quantified in 5 dpf zebrafish larvae for this study.
PHS: primary head sinus.

Supplementary Table

Table S1: Primer sequences used in this study.

**qPCR Primer name**	**Sequence**
ef1a qPCR forward	CTGGAGGCCAGCTCAAACAT
ef1a qPCR reverse	ATCAAGAAGAGTAGTACCGCTAGCATTAC
flt4 qPCR forward	AAAGGGGAGACAACGACATG
flt4 qPCR reverse	CGGCACTAACGAGAAGAGAG
lyve1b qPCR forward	CAGCACAGTCTGTCTAGACG
lyve1b qPCR reverse	ACCAAACCTGCATGTCTGTAA
rpl13 qPCR forward	CATCTTGAGCTCCTCCTCAGTAC
rpl13 qPCR reverse	CATCTCTGTTGACTCACGTCG
vegfc qPCR forward	GGCCTCAACAGAGCTTCAAC
vegfc qPCR reverse	TCTCTTGGGGTCCACGTTAC
